# Supplementary material for: Efficacy of Phytochemicals Derived from Roots of Rondeletia odorata as Antioxidant, Antiulcer, Diuretic, Skin Brightening and Hemolytic Agents—A Comprehensive Biochemical and In Silico Study
Source: Molecules. 2022 Jun 30;27(13):4204. doi: 10.3390/molecules27134204 (PMC9268568; doi:10.3390/molecules27134204)
Supplement: Supplementary file 1 [file molecules-27-04204-s001.zip › molecules-1767971-supplementary.pdf]

Supplementary Materials

Efficacy of Phytochemicals Derived from Roots of *Rondeletia odorata* as Antioxidant, Antiulcer, Diuretic, Skin Brightening and Hemolytic Agents- A Comprehensive Biochemical and in Silico Study

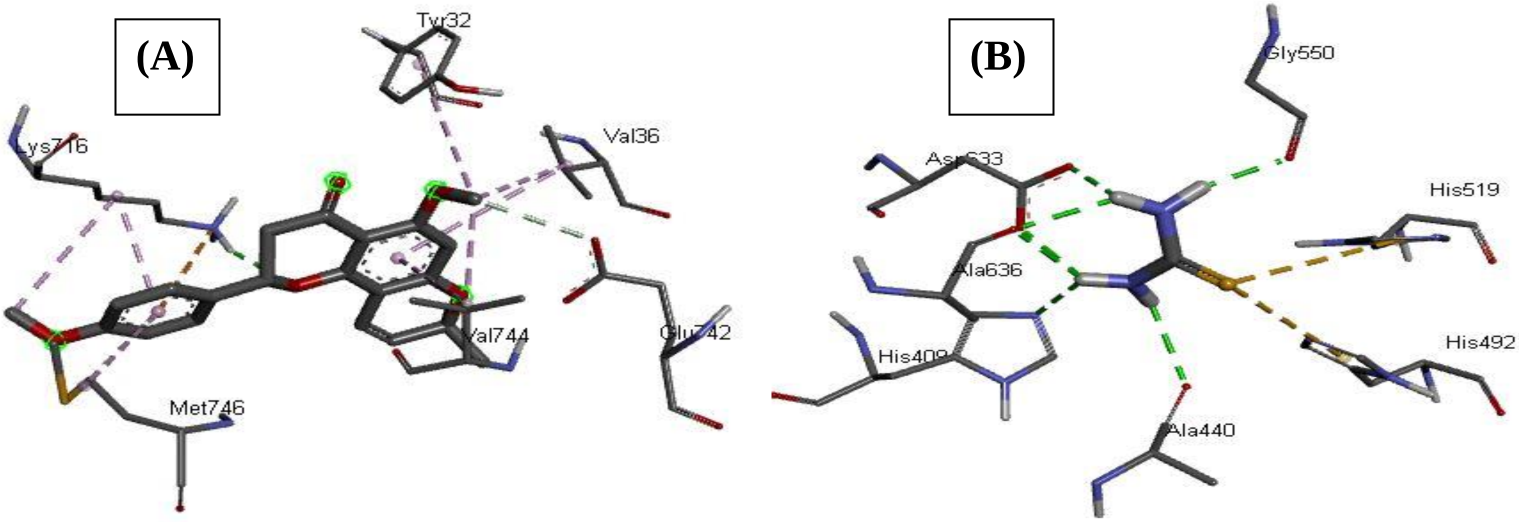

Figure S1. 3D structured binding affinities of **(A)** Glyflavanone A and **(B)** Thiourea (standard). with urease enzyme.

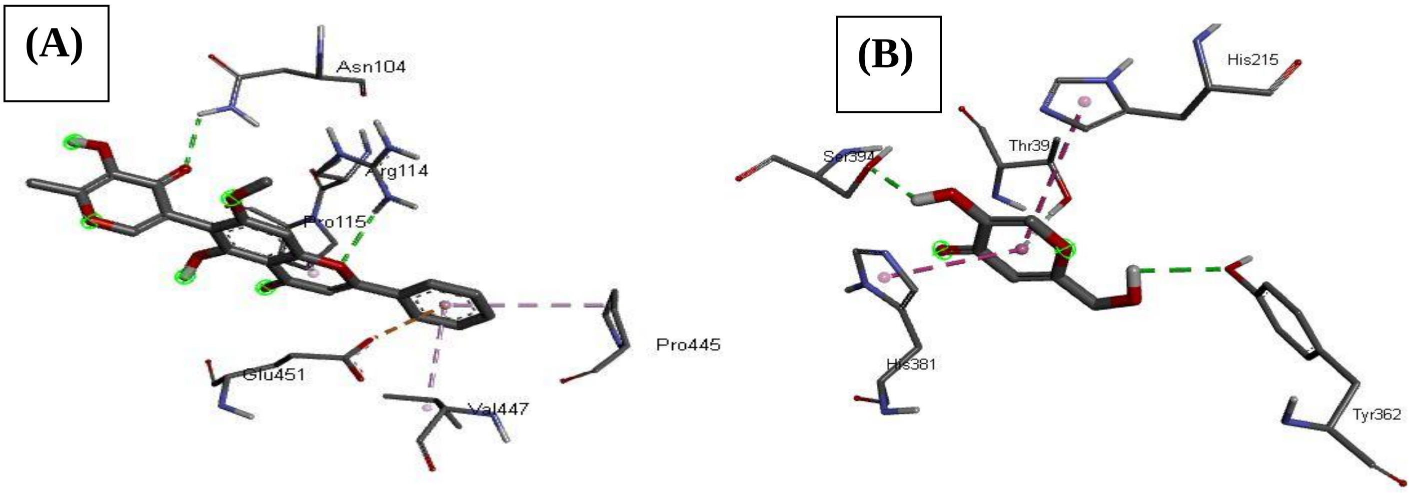

Figure S2. 3D Structured binding affinities of **(A)** Hosloppin and **(B)** Kojic acid with tyrosinase enzyme.

(A)

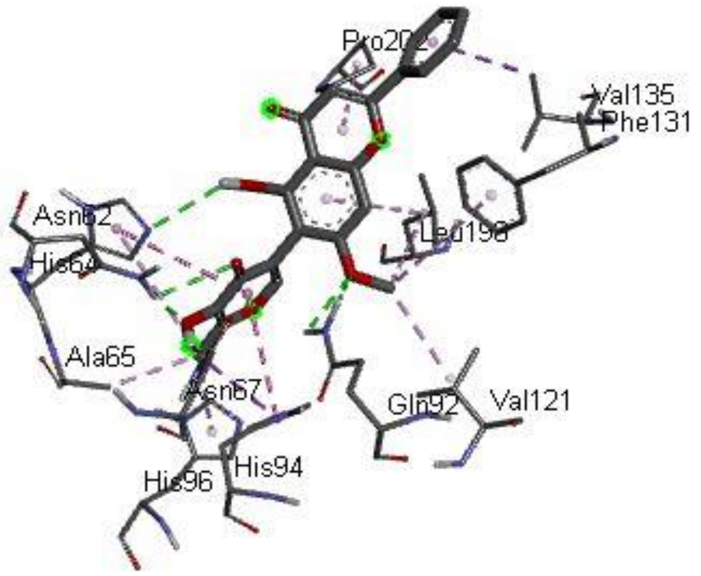

(B)

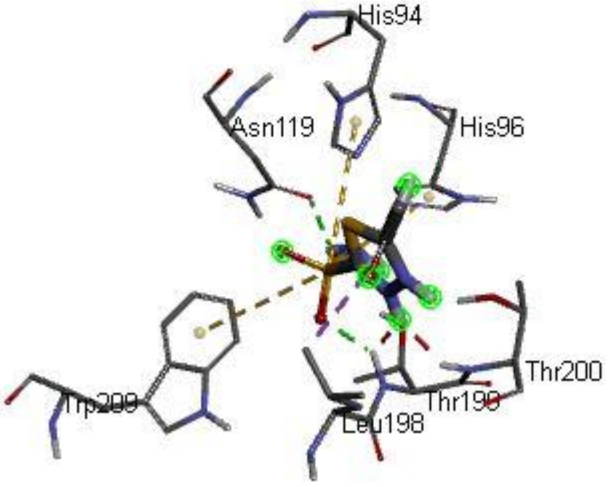

Figure S3. 3D structured binding affinities of (A) Hosloppin and (B) Acetazolamide with carbonic anhydrase enzyme.
